# Effective behavioral intervention strategies using mobile health applications for chronic disease management: a systematic review

**DOI:** 10.1186/s12911-018-0591-0

**Published:** 2018-02-20

**Authors:** Jung-Ah Lee, Mona Choi, Sang A Lee, Natalie Jiang

**Affiliations:** 1 Sue and Bill Gross School of Nursing, University of California Irvine, Irvine, CA USA; 2 College of Nursing, Mo-Im Kim Nursing Research Institute, Yonsei University, 50 Yonsei-ro, Seodaemun-gu, Seoul, Republic of Korea 03722; 3 College of Nursing and Health Sciences, University of Massachusetts, Boston, MA USA; 4 Program in Public Health, University of California Irvine, Irvine, CA USA

**Keywords:** Mobile applications, Disease management, Mobile health, Chronic disease management, Self-management

## Abstract

**Background:**

Mobile health (mHealth) has continuously been used as a method in behavioral research to improve self-management in patients with chronic diseases. However, the evidence of its effectiveness in chronic disease management in the adult population is still lacking. We conducted a systematic review to examine the effectiveness of mHealth interventions on process measures as well as health outcomes in randomized controlled trials (RCTs) to improve chronic disease management.

**Methods:**

Relevant randomized controlled studies that were published between January 2005 and March 2016 were searched in six databases: PubMed, CINAHL, EMBASE, the Cochrane Library, PsycINFO, and Web of Science. The inclusion criteria were RCTs that conducted an intervention using mobile devices such as smartphones or tablets for adult patients with chronic diseases to examine disease management or health promotion.

**Results:**

Of the 12 RCTs reviewed, 10 of the mHealth interventions demonstrated statistically significant improvement in some health outcomes. The most common features of mHealth systems used in the reviewed RCTs were real-time or regular basis symptom assessments, pre-programed reminders, or feedbacks tailored specifically to the data provided by participants via mHealth devices. Most studies developed their own mHealth systems including mobile apps. Training of mHealth systems was provided to participants in person or through paper-based instructions. None of the studies reported the relationship between health outcomes and patient engagement levels on the mHealth system.

**Conclusions:**

Findings from mHealth intervention studies for chronic disease management have shown promising aspects, particularly in improving self-management and some health outcomes.

**Electronic supplementary material:**

The online version of this article (10.1186/s12911-018-0591-0) contains supplementary material, which is available to authorized users.

## Background

The prevalence of chronic diseases, such as cancer, cardiovascular diseases, chronic pain, diabetes, and respiratory diseases is continuously increasing with regard to an aging society worldwide. According to the World Health Organization, chronic diseases are the leading cause of mortality in the world, accounting for more than 60% of all deaths [1]. Chronic disease is, therefore, a global burden. For example, according to the report by the Centers for Disease Control and Prevention (CDC) in the United States (US), about half of all American adults, approximately 117 million people, have one or more chronic disease conditions including heart disease, stroke, cancer, type 2 diabetes, obesity, or arthritis [2]. One in four adults in the US had two or more chronic diseases in 2012 [2]. Chronic diseases are the main cause of death among Americans, with 48% dying from cancer or heart diseases in 2010 [3]. In 2010, about 86% of Americans’ health care expenditure was for chronic disease treatment [4]. Therefore, chronic disease management is now a major public health issue in the US. Likewise, managing chronic diseases is also a challenge in other countries [5, 6]. For instance, over 40% of the population aged 15 years or older had a chronic disease condition in the European Union countries [5] and chronic diseases accounted for a substantial proportion of deaths throughout Southeast Asia [6].

With advances in mobile technologies, approaches based on mobile health (mHealth)—defined as “an area of electronic health (eHealth) with the provision of health services and information via mobile technologies such as mobile phones and Personal Digital Assistants (PDAs)” [7]—have been very popular in health care and public health [8–11]. Current evidence shows that the advantages of using mHealth devices are not only for the improvement of diagnosis and treatment but also the social connection with people [12]. Behavioral interventions using mobile applications (apps) on smartphones or tablet computers in enhancing self-management for patients with chronic diseases, such as heart failure [13] or diabetes [14], have been studied [9, 12]. For instance, a food intake diary, physical activity monitoring, and home blood sugar monitoring via mHealth systems are commonly used for diabetes management [14–16] while monitoring of weight, symptoms, and physical activity are common features of heart failure interventions [13, 17].

However, the evidence from current literature using the mHealth approach on improving health outcomes is inconsistent; some studies have shown that mHealth-based behavioral interventions are potentially effective in chronic disease management, whereas other studies did not obtain supportive results [9]. Previously, the evaluation of mHealth-based research focused on feasibility and acceptability of mHealth tools.

Rather than relying on feasibility research, which often does not utilize randomization in their intervention and/or a control group, and often lacks an effective size, a number of systematic or integrated reviews examined randomized controlled trials (RCTs) in diabetes management. These studies have demonstrated positive physiological and behavioral outcomes as well as incentive driven outcomes with mHealth systems [14–17]. However, there is limited literature showing that mHealth approaches can be useful for the self-management in patients with other chronic diseases. Therefore, an in-depth evaluation of RCTs on interventions that employ mHealth technologies and participants’ adherence to the interventions, training methods, intervention dosage, and length of follow-ups as outcomes of interest should be performed to provide recommendations on what factors make mHealth interventions effective for chronic disease management.

Thus, the purpose of this study was to perform a systematic review of RCTs using mHealth interventions for chronic disease management in adult populations to examine the effectiveness of mHealth interventions on health outcomes and process measures.

## Methods

The Preferred Reporting Items for Systematic Review and Meta-Analysis (PRISMA) guidelines [18] were used in this systematic review. The PICOS (participants, interventions, comparisons, outcomes, and study design) approach was used to develop a research question to guide the search strategies and review: that is, do interventions using mobile health applications improve health outcomes and process measures for adults with chronic diseases in RCTs?

### Search strategies

Searches were performed to retrieve studies that were published in peer-reviewed journals from January 2005 to March 2016, and written in English; the following databases were used: PubMed, CINAHL, EMBASE, the Cochrane Library, PsycINFO, and Web of Science. We used combinations of the key words and indexing terms such as MeSH or Emtree linked to the search domains. An example of a PubMed search strategy is as follows: for mobile interventions, “Mobile Applications”[Mesh] OR “Cell Phones”[Mesh] OR “Computers, Handheld”[Mesh] OR “mobile health” OR “m-health” OR mhealth OR “mobile-health” OR smartphone* OR “smart-phone*” OR “mobile phone*” OR “mobile-phone*” OR “cellular phone*” OR “cellular-phone*” OR “smart device*” OR “smart-device*” OR “tablet* PC*” OR “tablet-based” OR “tablet* device*”; for chronic disease outcomes, “Disease Management”[Mesh] OR “Chronic Disease/prevention and control”[Mesh] OR “Chronic Disease/therapy”[Mesh] OR “disease* manag*” OR “disease* monitor*” OR monitor* OR “health promot*” OR Promot*, and for method, “Randomized Controlled Trial” [Publication Type] OR “Randomized Controlled Trials as Topic”[Mesh] OR “Controlled Clinical Trial”[Publication Type] OR randomized[Title/Abstract] OR randomised[Title/Abstract] OR randomly[Title/Abstract] OR “random* assign*”[Title/Abstract] OR trial*[Title/Abstract]. Then, those three groups of search results were combined with “AND” (see Additional file 1 for search strategy for each database).

### Study selection

The inclusion criteria were as follows: adult patients with chronic diseases (except diabetes) as the target population, an intervention that involved using a mobile application for smartphones or tablets, and assessing the health outcomes and process measures. The exclusion criteria were as follows: studies that focused on a healthy population, pregnant women, non-adults (i.e., adolescents and children), or healthcare providers (e.g., apps for physicians’ or nurses’ use only); studies that used only qualitative methods (e.g., focus groups or group/individual interviews) or simple usability tests; and studies that measured psychological outcomes only (Table 1). Two reviewers (MC and SAL) independently screened titles, abstracts, and full-text articles to decide whether an article was relevant to the review. In case of disagreement, a third person was consulted (JL). We excluded studies of diabetes management because several systematic reviews and integrated reviews have already been published to report the effectiveness of mHealth-based interventions [14–17]. Only studies published in peer-reviewed journals were included.

**Table 1:** Inclusion and exclusion criteria

Inclusion criteria	• studies that included adult patients with chronic diseases (except diabetes) as the target population • studies that involved using a mobile application • studies that focused on disease management or health promotion
Exclusion criteria	• studies that included healthy people, pregnant women, non-adults (i.e., adolescents and children), and healthcare providers • studies that used only qualitative methods or simple usability tests • studies that measured psychological outcomes only

### Data extraction

Data were extracted from the selected articles and entered into an electronic data sheet. The contents of the data sheet included year of publication, research question or purpose, study design, types of disease, types of outcome and measurement, and the main results. In instances of disagreement, each case was discussed by the authors.

### Assessment of risk of bias

Selection bias (random sequence generation and allocation concealment), performance bias (blinding of participants and personnel), detection bias (blinding of outcome assessment), attrition bias (incomplete outcome data), reporting bias (selective reporting), and other biases (determined according to sample size calculation method, inclusion/exclusion criteria for patients’ recruitment, comparability of baseline data, funding sources, and any other potential methodological flaw that might have influenced the overall assessment) were assessed with the tool for risk of bias given in the Cochrane Handbook for Systematic Reviews of Intervention [19]. For each risk of bias item, the studies were classified as “unclear,” “low,” or “high” risk of bias respectively. Two reviewers (MC and SAL) assessed the trials independently and disagreements between two authors were resolved via discussion.

### Operational definitions of the terms used in this review

Studies on feasibility assess whether or not an intervention is appropriate for further testing, whereas studies on acceptability (which is a component of feasibility) determine how recipients react to that intervention [20]. Effectiveness is defined as an intervention study that shows statistical differences of one or more outcomes of interest measured between intervention and control groups. Health outcomes included physiological outcomes (e.g., gait and balance in patients with Parkinson’s disease or fatigue in patients with cancer) and psychological outcomes (e.g., quality of life, depressive symptoms, anxiety). Process measures [21] included participants’ adherence to, satisfaction with, and/or the level of engagement with mHealth systems. These process measures could be assessed via quantitative tools such as surveys or qualitative methods such as open-ended questions or a focus group interviews with intervention participants.

## Results

**Fig. 1:**
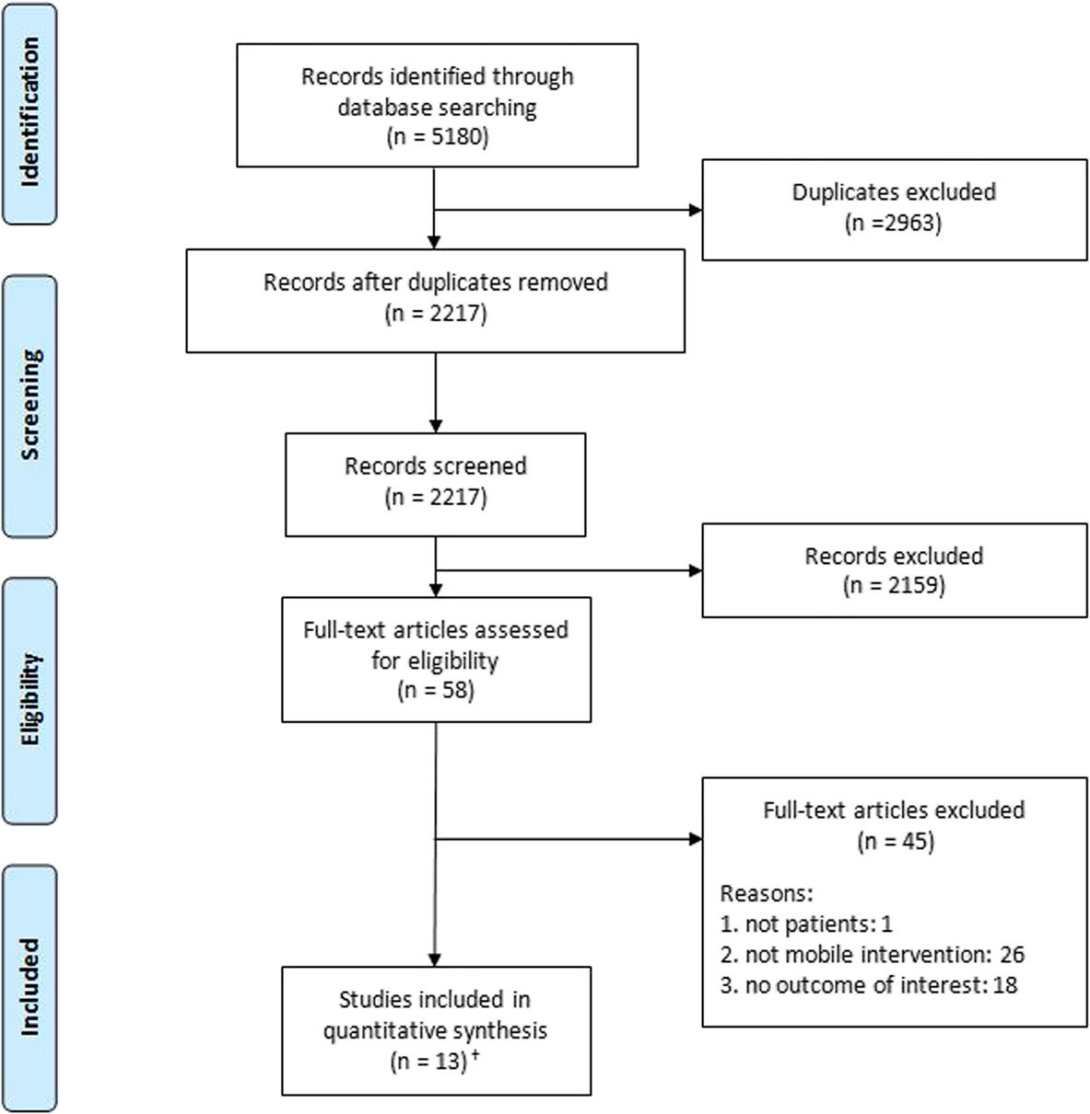
PRISMA flow diagram for the systematic review process. The step-by-step process of the application of inclusion and exclusion criteria generated the final number of studies included in the systematic review. ^†^Note: Kristjánsdóttir et al. (2013) was published as part 1 [22] and part 2 [23] with respect to a short-term follow-up and long-term follow-up; thus, the results are based on 12 studies from 13 published articles

Figure 1 shows the PRISMA flow diagram indicating the search process to select the final studies that met the inclusion criteria and thus were included in this systematic review. A study conducted by Kristjánsdóttir et al. was published as part 1 [22] and part 2 [23], which corresponded to short-term and long-term follow-ups, respectively. The results of both follow-ups were reviewed. Accordingly, the results of this systematic review are based on 12 studies from 13 published articles with quantitative evaluations.

Table 2 presents the summary of 12 RCT studies reviewed in this paper. The variety of chronic diseases managed using mobile apps included allergic rhinitis and asthma, cancer, cardiovascular diseases, chronic pain, chronic kidney disease, lung transplantation, Parkinson’s disease (PD), and spinal bifida. Diabetes management using mHealth apps has been evaluated in other literature and thus was not included. Among the 12 studies reviewed, 10 studies showed statistically significant mHealth app intervention effects on some variables that were examined in each study, while two of the 12 studies reviewed showed no statistically significant mHealth intervention effects on outcomes of interest when comparing between treatment and control groups. Two studies in this review were feasibility studies aimed at testing mHealth interventions for chronic disease management [24, 25] and one study was a pilot study evaluating a smartphone-based symptom management system for chemotherapy management [26]. These studies were also listed as RCTs.

**Table 2:** Summary of studies reviewed

Year/Author/Country	Purpose of Study	Sample	Types of Disease	Types of Outcomes and Measurements	Main Results
2009 Kearney et al. United Kingdom	To evaluate the impact of a mobile phone-based remote monitoring, advanced symptom management system (ASyMS©) on the incidence, severity and distress of six chemotherapy-related symptoms in patients with lung, breast, or colorectal cancer.	*n* = 112 (56 in each intervention or control group) patients from 7 clinical sites throughout the UK. Inclusion criteria: commencing a new course of chemotherapy treatment, receiving outpatient chemotherapy, age ≥ 18, written informed consent given, able to read and write English, and deemed by members of the clinical team as being physically and psychologically fit to participate in the study.	Chemotherapy related toxicity in patients with lung, breast, or colorectal cancer	• Severity and distress of the six symptoms including vomiting, nausea, diarrhea, hand-foot syndrome, sore mouth/throat, and fatigue. • Incidence – (did symptom occur? Y/N), Severity and distress (scores 0–3) of the six individual symptoms. • ASyMS has integrated the Common Toxicity Criteria Adverse Events (CTCAE) grading system and the Chemotherapy Symptom Assessment Scale. • Paper version of the electronic symptom questionnaire was administrated at baseline, chemotherapy cycles 2, 3, 4, and 5 in both groups.	• Two of the six symptoms measured (fatigue and hand-foot syndrome) showed statistical significance between the control and intervention groups (respectively, *p* = 0.040, *p* = 0.031). • Patients reported improved communication with health professionals, improvements in the management of their symptoms, and feeling reassured their symptoms were being monitored while at home when using ASyMS.
2013 Kristjánsdóttir et al. Norway	To study the long term effects of a 4-week smartphone intervention with diaries and therapist feedback following an inpatient chronic pain rehabilitation program (11-month follow up of 2013 Kristjánsdóttir et al. study)	*n* = 135 (intervention group: 69/control group: 66) Inclusion criteria: female, age ≥ 18, participating in the inpatient multidimensional rehabilitation programfor chronic pain, having chronic widespread pain > 6 months (with or without diagnosis of fibromyalgia), not participating in another research project at the rehab center, being able to use a smartphone, and not being diagnosed with a profound psychiatric disorder.	Chronic widespread pain or Fibromyalgia	• Catastrophizing [Pain catastrophizing scale (PCS)] • Acceptance [Chronic pain acceptance questionnaire (CPAQ)] • Emotional distress [modified General Health Questionnaire (GHQ)] • Importance and success in living according to one’s own values in 6 domains (family, intimate relationships, friendship, work, health, and personal growth) [Chronic Pain Values Inventory (CPVI)] • Pain, fatigue, sleep disturbance [Visual analog scales (VAS)] • Impact of Fibromyalgia on functioning and symptom levels the past week [Fibromyalgia Impact Questionnaire (FIQ)] • Functioning [Short-Form Health Survey (SF-8)] • Use of noninteractive website [self-report at T3 (4 weeks after discharge)] • Feasibility of the smartphone intervention (single question for post-intervention)	Short-term follow-up results: • Intervention group reported less catastrophizing (*p* < 0.001). • Results from the per-protocol analysis indicate intervention with diaries and written personalized feedback reduced catastrophizing and increased acceptance and effects persisted 5 months after the intervention. • Increased improvement in values-based living in the intervention group • Control group showed an increased level of fatigue and a tendency toward an increase in sleep disturbance at the 5-month follow-up. Long-term 11-month follow-up results: • The between-group differences on catastrophizing, acceptance, functioning, and symptom level were no longer evident (*p* > 0.10). • More improvement in catastrophizing scores during the follow-up period (T2-T5) in the intervention group (*p* = 0.045) • Positive effect on acceptance was found within the intervention group (*p* < 0.001). • Small to large negative effects were found within the control group on functioning and symptom levels, emotional distress, and fatigue (*p* = 0.05). • Reduction in disease impact (measured by FIQ) found for intervention group (*p* = 0.03). • Long-term results are ambiguous.
2013 Garcia-Palacios et al. Spain	To compare compliance with paper diary vs. smartphone diary, aggregated ecological momentary assessment (EMA) data vs. retrospective data, and assess acceptability of EMA procedures.	*n* = 40 (intervention group:20/control group:20) Inclusion criteria: met criteria for FMS, defined by the American College of Rheumatology and were diagnosed by a rheumatologist.	Fibromyalgia syndrome (FMS)	• EMA pain and fatigue (0–10 Numerical Rating Scales) • Mood (face-based pictorial 7-point scale) • Weekly retrospective rating of pain and fatigue [Brief Pain Inventory (BPI) and Brief Fatigue Inventory (BFI)] • Acceptability and preferences (self-report)	• Smartphone condition (smartphone diary) showed higher levels of compliance than paper condition (paper diary) (*p* < 0.01). • Retrospective assessment produces overestimation of events (pain and fatigue, *p* < 0.01). • Smartphone condition preferred and accepted over paper diary, even in participants with low familiarity with technology.
2014 Vuorinen et al. Finland	To study whether multidisciplinary care with telemonitoring leads to decreased HF-related hospitalization	*n* = 94 (intervention group: 47/control group: 47) Inclusion criteria: diagnosis of systolic heart failure, age 18–90 years, NYHA (New Work Heart Association) functional class ≥2, left ventricular ejection fraction ≤35%, need for a regular check-up visit, and time from the last visit of less than 6 months.	Heart failure (HF)	• Number of HF-related hospital days (data from hospital electronic health record system) • Clinical effectiveness [death from any cause, heart transplant operation or listing for transplant operation, left ventricular ejection fraction (LVEF,%) measured by echocardiography, plasma concentration of N-terminal of the prohormone brain natriuretic peptide (NT-proBNP, ng/1), creatinine, sodium, and potassium] • Self-care behavior (European Heart Failure Self-Care Behavior Scale) • Use of health care resources (analyzed outpatient visits)	• No difference found in the number of HF-related hospital days (*p* = 0.351). • Intervention group used more health care resources. • No statistically significant differences in patients’ clinical health status or self-care behavior.
2015 Cingi et al. Turkey	To investigate the impact of a mobile patient engagement application on health outcomes and quality of life	*n* = 2282 interventions (physician on call patient engagement trial, POPET for patients with allergic rhinitis or asthma) POPET-AR (intervention group: 88/control group: 51) POPET-Asthma (intervention group: 60/control group:29)	Allergic rhinitis (AR) and asthma patients	• Health outcomes and quality of life [AR groups: Rhinitis Quality of Life Questionnaire (RQLQ), asthma groups: Asthma Control Test (ACT)]	• POPET-AR group showed better clinical improvement than the control group in terms of overall RQLQ score as well in measures of general problems, activity, symptoms other than nose/eye, and emotion domains (*p* < 0.05). • More patients in the POPET-Asthma group achieved a well-controlled asthma score compared to the control group (*p* < 0.05).
2015 Dicianno et al. United States	To determine feasibility of the interactive mobile health and rehabilitation (iMHere) system and its effects on psychosocial and medical outcomes	*n* = 23 (intervention group:13/control group:10) Inclusion criteria: age 18–40, primary diagnosis of myelomeningocele with hydrocephalus, ability to use smartphone, and living within 100 miles of testing site to allow for technical support.	Spina bifida (SB)	• Usage (the number of participant responses to reminders, use of secure messaging, or photo uploads) • Physical independence (Craig Handicap Assessment and Reporting Technique Short Form, Physical independence domain) • Self management skill (Adolescent Self-Management and Independence Scale II) • Depressive symptoms (The Beck Depression Inventory-II) • Perception of patient-centered care (Patient Assessment of Chronic Illness Care) • Quality of Life (World Health Organization Quality of Life Brief Instrument) • Number of UTIs (diagnosed UTIs) • Number of wounds (unique skin breakdown episodes that were at least stage II) • Number of emergency department (ED) visits (ED visits for any reason) • Number of ED visits due to UTI or wound • Number of planned and unplanned hospitalizations • Number of hospitalizations due to UTI or wound	• Smartphone system was found to be feasible and associated with short-term self-reported improvements in self-management skills.
2015 Hägglund et al. Sweden	To evaluate whether a home intervention system (HIS) using a tablet had an effect on self-care behavior.	*n* = 82 (intervention group:42/control group:40) Inclusion criteria: hospitalized and diagnosed for HF with reduced ejection fraction (HFrEF) and/or preserved EF (HFpEF), treatment with diuretics, and referred straight to primary care.	Heart failure (HF)	• Disease-specific self-care (European Heart Failure Self-Care Behavior Scale) • Health-related quality of life (HRQoL) (Kansas City Cardiomyopathy Questionnaire) • Adherence (frequency of HIS use) • Knowledge (Dutch Heart Failure Knowledge Scale) • HF-related hospital days (patients’ case books)	• Intervention group showed improvement in self-care and HRQoL, reduction in HF-related hospital days.
2015 Martin et al. United States	To investigate whether a fully automated mHealth intervention with tracking and texting components increases physical activity.	*n* = 48 [unblinded = 32 (smart texts = 16, no texts = 16), blinded = 16] Unblinded participants were randomized to smart texts or no texts in phase II (weeks 4–5). Inclusion criteria: ages 18–69, using a Fitbug compatible smartphone (iPhone≥4S, Galaxy≥S3).	Cardiovascular disease (CVD)	• Mean change in accelerometer-measured daily step count (measured by Fitbug Orb) • Attainment of prescribed 10,000 steps/day goal (measured by Fitbug Orb) • Changes in total daily activity and aerobic time (measured by Fitbug Orb)	• Intervention with texting component increased physical activity (*p* < 0.001).
2015 Piette et al. United States	To compare the effects of systematic feedback to HF patients’ caregivers and HF patients receiving standard mHealth.	*n* = 372 (intervention group:189/control group: 183) Inclusion criteria: HF diagnosis, ejection fraction < 40%, able to name eligible CarePartner (CP) that is a relative or friend living outside their home.	Heart failure (HF)	• HF-related quality of life (Minnesota Living with Heart Failure Questionnaire) • Patient-CP communication (quantitative telephone surveys) • Medication adherence and self-care (Revised Heart Failure Self-Care Behavior Scale)	• mHealth + CP (intervention) group showed improvement in medication adherence and caregiver communication. • mHealth + CP may improve qualify of life in patients with greater depressive symptoms and also decrease patients’ risk of shortness of breath and sudden weight gains.
2016 Cubo et al. Spain	To evaluate the cost-effectiveness of home-based motor monitoring (HBMM) with in-office visits versus in-office visits alone in patients with advanced Parkinson’s disease	n = 40 (intervention group: 20/control group: 20) Inclusion criteria: non-demented outpatients from a tertiary regional movement disorders clinic, Mini-Mental Scale score > 24, and diagnosed with idiopathic, advanced PD.	Parkinson’s disease (PD)	• Motor (Unified Parkinson’s Disease Rating Scale and Hoehn and Yahr staging Scale) and non-motor (Non-Motor Symptoms Questionnaire Scale) symptom severities • Cost-effectiveness (incremental cost-effectiveness ratio) • Direct costs (standardized questionnaire) • Quality of life (EuroQoL) • Neuropsychiatric symptoms (Hospital Anxiety Depression Scale, Scale for Evaluation of Neuropsychiatric Disorders, Parkinson Psychiatric Rating Scale) • Comorbidities (Cumulative Illness Rating scale-Geriatric)	• HBMM was found to be cost-effective in improvement of functional status, motor severity, and motor complications.
2016 DeVito Dabbs et al. United States	To compare the efficacy of an mHealth intervention in promoting self-management behaviors and self-care agency, rehospitalization, and mortality at home during the first year after lung transplantation.	*n* = 201 (intervention group: 99/control group: 102) Inclusion criteria: age > 18, received transplantation at the University of Pittsburgh Medical Center, and could read and speak English.	Lung transplant recipients (LTRs)	• Self-monitoring (percentage of days that LTRs performed self-monitoring) • Adherence to regimen (Health Habits Survey) • Critical health (percentage of critical indicators) • Self-care agency (Perception of Self-Care Agency) • Health outcomes (medical records)	• The intervention group performed self-monitoring (*p* < 0.001), adhered to medical regimen. (*p* = 0.046), and reported abnormal health indicators (*p* < 0.001) more frequently. Than the usual care group. • Both groups did not differ in re-hospitalization (*p* = 0.51) or mortality (*p* = 0.25).
2016 Ginis et al. Israel and Belgium	To determine the feasibility and effectiveness of the gait training CuPiD-system for people with Parkinson’s disease in the home environment.	n = 40 (intervention group: 22/control group: 18) Inclusion criteria: ability to walk 0 min continuously, score of ≥24 on Montreal Cognitive Assessment, Hoehn and Yahr Stage II to III in ON-state, and on stable PD medication.	Parkinson’s disease (PD)	• Single and dual task gait (gait speed) • Balance (mini-Balance Evaluation Systems Test, Four Square Step Test, Falls Efficacy Scale-International) • Endurance and physical capacity (2 Minute Walk Test, Physical Activity Scale for the Elderly) • Disease severity (Movement Disorders Unified Parkinson’s Disease Rating Scale – motor examination) • Freezing of gait (New FOG Questionnaire, Ziegler protocol) • Cognition (Color Trail Test A & B, sitting & walking verbal fluency) Quality of life (Short Form 36 Health Survey)	The CuPiD-system was feasible and effective, as the intervention group improved significantly more on balance and maintained quality of life compared to the control group.

### Studies reporting significant effects on outcomes

The majority of mHealth RCT-studies in this systematic review (10 out of 12 studies, 83.3%) showed statistically significant effects on health outcomes by incorporating mobile applications in managing chronic diseases. Those studies demonstrated improved physical functioning, adherence to prescribed medications, and/or ease of symptom evaluation and reports to care providers, as well as process measures including patient satisfaction with mHealth management and feasibility of smart-phone-based self-management interventions.

Kearney et al. [26] in the United Kingdom reported significant improvement in fatigue (odds ratio, OR = 2.29; 95% CI, 1.04–5.05; *P* = 0.040) and hand-foot syndrome (OR control/intervention =0.39; 95% CI, 0.17–0.92; *P* = 0.031) in patients with lung, breast, and colorectal cancer using a mobile phone-based remote monitoring of chemotherapy-related symptoms in comparison to the usual care group. In Norway, Kristjánsdóttir et al. [22, 23] showed a favorable effect on pain management in a 4-week follow-up (catastrophizing score lower for *Intervention, M* = 9.20, SD = 5.85, compared to *Control, M* = 15.71, SD = 9.22, *P* < 0.01, with a large effect size, Cohen’s *d* = 0.87) but not in the 5-month and 11-month follow-ups (outcome variables including catastrophizing, acceptance, functioning, and symptom level, all *P* > 0.1). In Spain, Garcia-Palacios et al. [27] developed an ecological momentary assessment (EMA) for chronic pain in fibromyalgia patients and found that patients with less familiarity with technology using a mobile EMA system via their smartphones showed higher levels of compliance than patients with a paper-based diary (complete record *t* = − 4.446, *d* = 1.02, reference Cohen’s *d* > =0.8, large effect). In Turkey, Cingi et al. [28] reported that patients with allergic rhinitis or asthma displayed better quality of life or well-controlled asthma scores by using the mHealth intervention compared to the control group (all *P* < 0.05). Dicianno et al. [24] demonstrated the feasibility of a mHealth intervention for patients with spina bifida to improve self-management skills and high usage of the mobile system was associated with positive changes in the self-management skills. In Sweden, Hägglund et al. [29] tested a tablet-based intervention in patients with heart failure (HF) and found improved self-care and health-related quality of life (HRQoL) and a reduction in HF-related hospital days (risk ratio, RR = 0.38; 95% CI, 0.31–0.46; *P* < 0.05). In Israel, Ginis et al. [25] conducted home-based smartphone-delivery automated feedback training for gait in people with PD, and found significant improvement in balance (*F*_(2,108)_ = 3.73, *P* = 0.04) from baseline to post-test.

Martin et al. [30] in the US found that an automated text message system increased physical activity to prevent cardiovascular diseases in phase 1 (weeks 2 to 3) and phase 2 (weeks 4 to 5), all *P* < 0.001. Piette et al. [31] in the US reported that the intervention group involving “CarePartners” connecting to a relative or friend living outside their home showed improvement in medication adherence and caregiver communication (all *P* < 0.05). DeVito Dabbs et al. [32] in the US conducted an RCT for patients with lung transplantation and reported improvement in self-monitoring (OR = 5.11; 95% CI, 2.95–8.87; *P* < 0.001), adherence to medical regimen (OR =1.64; 95% CI, 1.01–2.66, *P* = 0.046), and reported abnormal health indicators more frequently (OR = 8.9; 95% CI, 3.60–21.99; *P* < 0.001).

### Studies reporting similar or no effects on outcomes

Two of the twelve studies reviewed (16.7%) showed similar or no effects of mHealth-based interventions on main outcomes of interest compared to control groups. In Finland, Vuorinen et al. [33] found no difference in the number of HF-related hospital days (incidence rate ratio, IRR = 0.812, *P* = 0.351). However, patients in the telemonitoring intervention group used more healthcare resources; increased number of visits to the nurse (IRR = 1.73; 95% CI, 1.38–2.15; *P* < 0.001), more time spent with nurses (mean difference = 48.7 min, *P* < 0.001), and increased number of telephone contacts initiated by nurses (IRR = 5.6; 95% CI, 3.41–7.63; *P* < 0.001). In Spain, Cubo et al. [34] reported a trend of lower PD functional status (the Unified Parkinson’s Disease Rating Scale, UPDRS I) in patients on home-based monitoring compared to patients in standard in-office visits (*P* = 0.06), while other outcomes (measured by UPDRS II, III, IV subscales) and HRQoL in PD did not show statistically significant differences between the intervention and control groups. Cubo et al. [34], however, explained that the approach of using home-based motor monitoring via mHealth applications compared to standard office visits in the 1-year follow up was cost-effective (incremental cost-effectiveness ratio, ICER, per unit of UPDRS subscales ranging from €126.72 to € 701.31).

### mHealth interventions

Table 3 presents the details of mHealth interventions including duration, mobile app type, app content, and training methods. A quarter of the studies (3 out of 12) reported the feasibility of the mHealth intervention as a pilot study to assess the potential for successful implementation of the mHealth intervention to patients/participants [24–26]. The majority of studies used smartphones as a mobile device, two studies [29, 34] used tablets for mHealth interventions, and two studies used telemonitoring wireless devices including weight scales for patients with HF [29] or gait detectors for patients with PD [25]. The length of interventions ranged from two weeks to twelve months; half of the studies (6/12, 50%) had more than six-month intervention periods and follow-ups. Most common components of mHealth interventions included remote symptom monitoring and self-assessment as well as tailored automated messages or self-care education to coach patients with chronic disease conditions that needed active disease management. One particular study by Kearney et al. was more inclusive in that it provided real-time feedback and tailored such feedback for symptom management depending on the severity, offering pharmacological, nutritional, or behavioral advice when needed [26].

**Table 3:** Details of mobile application intervention for chronic disease management

Study	Length of Intervention	Name of Mobile Application and Platform	Program of Intervention	Delivery of Intervention (Training of mHealth)
2009 Kearney et al. United Kingdom	Five time pointes (baseline, chemotherapy cycle 2, 3, 4, and; each cycle has up to 14 days).	Advanced symptom management system (ASyMS©)	• A mobile phone-based remote monitoring and reporting of chemotherapy-related toxicity. • Participants completed the electronic symptom questionnaire on their mobile phone, took their temperature using an electronic thermometer and entered the value into the application twice a day for 2 weeks after their first 4 cycles of chemotherapy • Patients received tailored self-care advice on their mobile phone based on the severity of symptoms reported	Patients were trained on how to use the system by nurses working in their local clinic who had received training by the study team on how to use the system.
2013 Kristjánsdóttir et al. Norway	4 weeks	Application: Diaries and Daily Situational Feedback Smartphone: HTC TyTN (touchscreen and keyboard)	• The intervention consisted of 4 components: face-to-face session – 1 h individual session with nurse, web-based diaries – 3 diary entries/day using the smartphone, written situational feedback – daily written feedback from therapist on information provided in diary, and audio files – 4 mindfulness exercises guided by the authors • All participants received access to a non-interactive website with information on self-management strategies for people with chronic pain • Self-reported assessments on paper were gathered before (T1) and after (T2) the inpatient program, immediately after the smartphone intervention which was 4 weeks after discharge (T3), and 5 (T4) and 11 months (T5) after the smartphone intervention	Patients attended an informational group meeting. Participants were lent smartphones and received information about their therapist for the intervention during the face-to-face session.
2013 Garcia-Palacios et al. Spain	2 weeks	Software application: F-EMA (ecological momentary assessment) Smartphone: HTC Diamond 1 (TOUCH Diamond 1, HTC Corporation, New Taipei City, Taiwan) Software: Windows Mobile 6.1	• Session 1 (7 days): participants were randomly assigned a self-record condition and recorded their pain, fatigue, and mood 3 times/day • Session 2 (7 days): acceptability questionnaire and Brief Pain Inventory (BPI) and Brief Fatigue inventory (BFI) were administered regarding the first condition, and participants received the other self-record condition • Session 3: acceptability questionnaire, BPI and BFI, and preference questionnaire were administered regarding the second condition	Participants attended an individual information session during the first week. They were given verbal instructions on the self-record method, explanations of the scales, and practiced rating the scales with the researcher. An information sheet with definitions of each scale and instructions for the self-recording were given to each patient.
2014 Vuorinen et al. Finland	6 months	Application name not available. Application enabled recording of all necessary measurements and symptoms.	• Patient made measurements (blood pressure, pulse, and body weight), assessed symptoms (dizziness, dyspnea, palpitation, weakness, and edema), and evaluated overall condition (deteriorated, improved, or remained unchanged) once a week • Patient received automatic machine-based feedback of whether parameter was within personal targets set by nurse • Nurse contacted patient each time measurement was beyond target levels	Patients given a home-care package: weight scale, blood pressure meter, mobile phone, and self-care instructions.
2015 Cingi et al. Turkey	1 month (patients with allergic rhinitis(AR)), 3 months (asthma patients)	Application: physician on call patient engagement trial (POPET-AR; POPET-Asthma)	• The application allowed patients to communicate with their physician, record their health status and medication compliance • Provided motivational and educational content • Reminded patients to take prescribed medications	Patients were educated on the recommended use of prescribed medications and informed about the Rhinitis Quality of Life Questionnaire and the Asthma Control Test. Trial information, application training, and technical support was available online.
2015 Dicianno et al. United States	12 months	Application: iMHere Smartphone: Android Provided participants with a phone plan that included unlimited texting and data.	• Intervention consisted of 6 modules, a web-based clinician portal, and a 2-way communication system • Modules served as reminders to perform various self-care tasks, record wounds, manage medications, complete mood surveys, and for secure messaging • Patient problems were triaged on a web-based dashboard for physicians	Participants were instructed to use the modules based on their own prescribed protocols.
2015 Hagglund et al. Sweden	3 months	Application: HIS: OPTILOGG Tablet wirelessly connected to weight scale.	• HIS monitored weight and symptoms, titrated diuretics, and provided information about HF and lifestyle advice	Intervention group received a basal information sheet. The HIS was installed in their home.
2015 Martin et al. United States	5 weeks	Smartphone application: Fitbug Digital physical activity tracker: Fitbug Orb Smartphone texting system: Reify	• Automated mHealth intervention with tracking and texting components • Unblinded participants could view their daily step count, activity time, and aerobic activity time through smartphone and web interfaces; blinded participants were unable to view this information • Smart texts delivered coaching 3 times/day aimed at individual encouragement and fostering feedback loops by an automated, physician written, theory-based algorithm with a goal of 10,00 steps/day	No training mentioned.
2015 Piette et al. United States	12 months	mHealth application was not mentioned. Intervention used interactive voice response (IVR) telephone calls.	• Standard mHealth group received weekly interactive voice response (IVR) calls with tailored self-management advice • mHealth + CarePartner (CP) group received the same intervention but with automated emails sent to their CP after each IVR call with feedback about the heart failure (HF) patient’s status and suggestions to support disease care • CP called their patient-partner weekly to review reports and address identified problems	Both groups were mailed information about HF self-care. CPs received guidelines about how to communicate in a positive motivating way, avoid conflict by respecting boundaries, include in-home caregivers, and respect confidentiality.
2016 Cubo et al. Spain	12 months	System: Kinesia, included tablet software app, wireless finger-worn motion sensor unit, and automated web-based symptom reporting.	• All patients with Parkinson’s disease (PD) completed structured questionnaires and were assessed under the beneficial effect of the antiparkinsonian drugs in the clinic every 4 months following the same protocol • PD motor symptoms were monitored at home 1 day/month with 3–6 motor assessments and a structured questionnaire in the HBMM group	Assistant brought Kinesia device and provided training in each participant’s home.
2016 DeVito Dabbs et al. United States	12 months	Program: Pocket Personal Assistant for Tracking Health (Pocket PATH)	• Participants recorded daily health indicators, viewed graphical displays of trends, and received automatic feedback messages when reaching critical threshold values using the Pocket PATH system	Patients received scripted discharge instructions from an interventionist and an instruction binder emphasizing the importance of performing daily self-management behaviors at home in 60 min training sessions.
2016 Ginis et al. Israel and Belgium	6 weeks	CuPiD-system: smartphone (Galaxy S3-mini, Samsung, South Korea), docking station, 2 inertial measurement units (EXLs3, EXEL srl., Italy), and 2 applications (the audio-bio feedback, ABF-gait app, the instrumented cueing for freezing of gait, FOG-cue app)	• The CuPiD-system measured gait in real-time, provided positive and corrective auditory biofeedback (ABF) on gait parameters, and rhythmical auditory cueing to prevent or overcome freezing of gait (FOG) episodes	Researchers provided gait training to CuPiD participants for 30 min, 3 times/week for 6 weeks. Participants with FOG were taught ways to avoid FOG and practiced for an additional 30 min, 3 times/week using the FOG-cue app. Pictures and personalized instructions were also given to participants. Telephone consultation was available for system support.

The sample size of the studies reviewed was between 28 (patients with spina bifida) and 372 (patients with heart failure). In terms of subjects, studies included patients who were 18 years and above, with some studies limited to a particular age range such as 18–40 [24] or up to 69 years [30]. The mean age of participants in the mHealth intervention studies ranged from 30-year-old patients with spina bifida [24] to 75-year-old patients with HF [29]; the approximate average age group in this 12-study review was in the 50s. None of the studies reported effectiveness of mHealth intervention by age categories. These 12 studies also did not report participants’ prior experience with mobile devices such as smartphones or educational background, which may have affected the ability to use mHealth apps via mobile devices. Outcomes of interventions were measured either by mHealth systems directly or by paper-based questionnaires.

In terms of mHealth intervention training, either face-to-face information sessions at baseline or paper-based instructions were used in most studies. Kearney et al. [26] found that symptoms were reported differently on a paper-based questionnaire and mobile phone. Participants in the mobile group reported lower levels of fatigue compared to those in the paper-based group (OR _non-mobile/mobile_ = 2.29, 95% CI 1.04–5.05, *P* = 0.04) [26]. Reporting cancer toxicity symptoms (e.g., hand-foot syndrome and mucositis) in real time might allow for more accurate measurement [26].

None of the studies reported process measures, including adherence, level of engagement, and/or satisfaction with the mHealth systems. Potentially, there was limited information on such measures in the published articles with the authors possibly publishing the process measures elsewhere.

### Risk of bias assessment

**Fig. 2:**
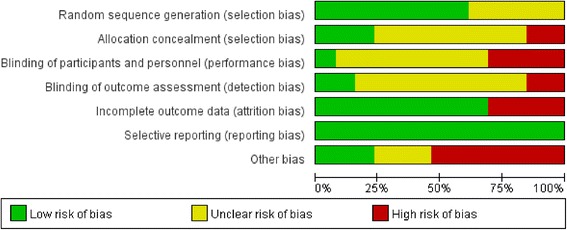
Risk of bias assessment: Summary graph

**Fig. 3:**
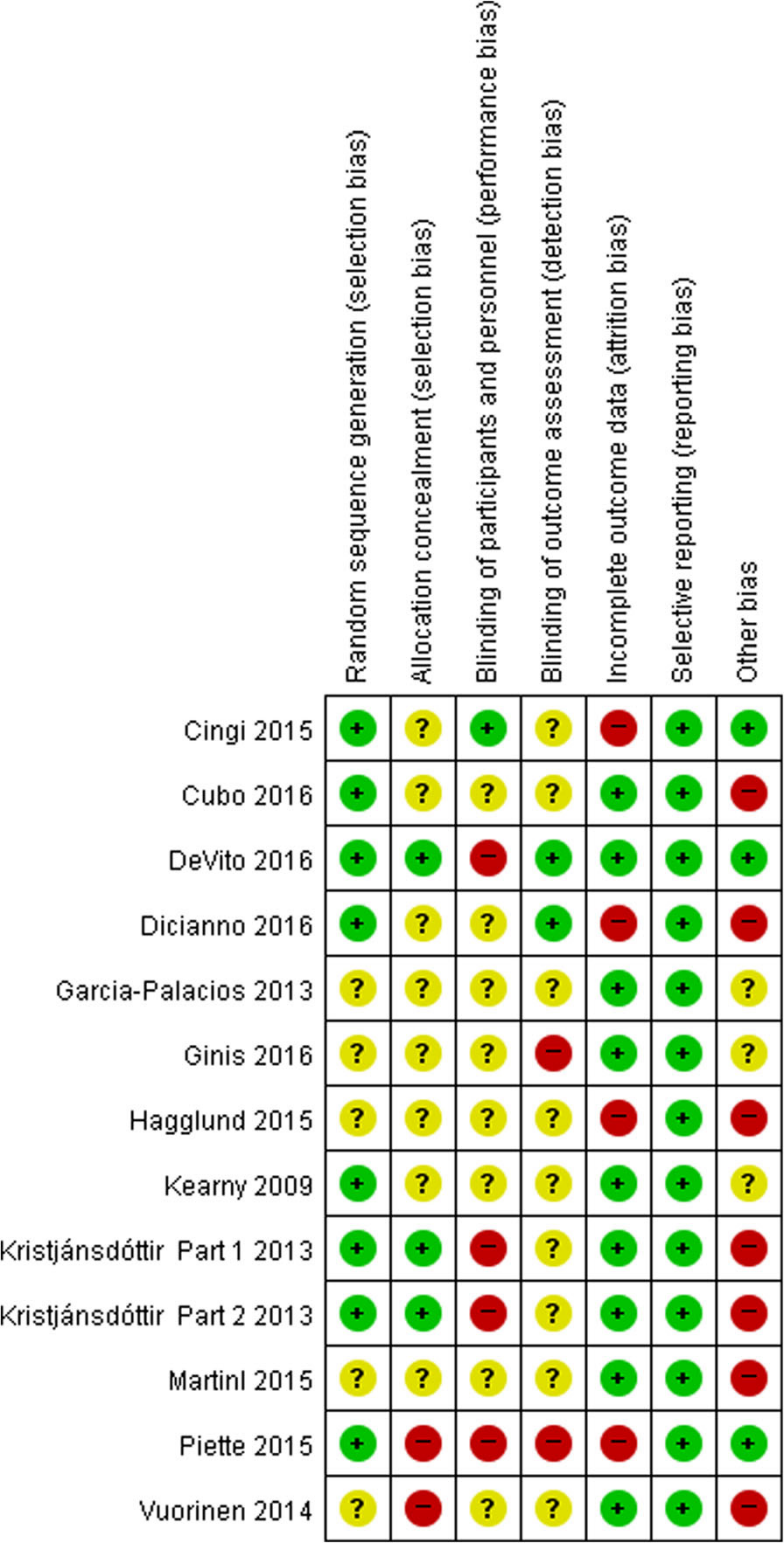
Risk of bias assessment for individual studies.  Low risk of bias;  Unclear;  High risk of bias

The risk of bias in the reviewed studies is summarized in Fig. 2 and shown for individual studies in Fig. 3. The intervention studies generally performed well in their risk of bias for random sequence generation (62% low risk), allocation concealment (23% low risk), blinding of participants and personnel (8% low risk), blinding of outcome assessors (15% low risk), incomplete outcome data (69% low risk), and selective reporting (92% low risk) (Fig. 2).

Considering the method of randomization, 5 trials did not present details of randomization [25, 27, 29, 30, 33]. Only 2 studies reported use of an adequate allocation concealment method [22, 32]. Although due to the nature of the mHealth intervention, it is almost impossible to blind participants and healthcare providers, one study described the blinding of participants by providing the same mobile phone application to both experimental and control groups with different functionalities. The experimental group was given communication, health status, or medication usage tracking, and a survey questionnaire, whereas the control group received only the survey questionnaire [28]. Only two trials were identified as blinding the outcome assessors. Four trials failed to provide a full description of participants and losses to follow-up during their trials [24, 28, 29, 31]. All studies had a low risk for reporting bias. Devito et al. had low risk for all items except blinding of participants and personnel [32]. Hägglund et al. had low risk only for reporting bias [29] (Fig. 3).

## Discussion

This systematic review has found a potential favorable effect of mHealth interventions on health outcomes and process measures in patients with chronic diseases including asthma, cancer, cardiovascular diseases, chronic pain, spina bifida, or Parkinson’s disease. The results from the reviewed RCTs showed improvement in some health outcomes in patients in managing their chronic disease.

There were some commonalities and differences in using mHealth in the reviewed studies. One of the common features useful in mHealth interventions is pre-set and tailored feedback on reported symptoms. The mobile application systems used in the reviewed studies were developed by the study team and validated and refined in the previous studies that were conducted before the RCTs. None of the commercial health apps were used in the reviewed studies. Most studies utilized research staff to provide training in mHealth systems for the participants via face-to-face or information group sessions, or through information materials, while one study [26] used local nurses to train patients. None of the reviewed studies addressed whether their mHealth systems were incorporated into daily medical practice in either clinic settings or acute care hospitals, meaning that there were no signs of their implementation in real health care systems. Challenges in real-life settings may relate to lack of financial incentives for providers in using mHealth tools or uncertainty regarding privacy and security of information transferred via mHealth systems [35].

Interventions to promote self-management in patients with chronic diseases started from web-based and/or telephone-based interventions to mHealth-based interventions. Unlike those previous behavioral interventions limited to places where patients with chronic diseases had access to the treatment advice, mHealth interventions have advanced features such as real-time symptom monitoring and feedback [25, 34, 36]. For example, patients with PD receive real-time feedback on their selected gait parameters during their walks via the preset gait app developed from evidence-based exercise guidelines [25]. This is an example of how using well-designed and validated mHealth apps in daily life can benefit health outcomes.

The number of smartphone users has shown great increases; in the US [37] it is estimated to reach 224.3 million in 2017, up from 171 million in 2014; worldwide it was [38] 2.32 billion in 2017, up from 1.57 billion in 2014. Approximately 77% of people in the US owned smartphones in 2016 [39]. Using mobile devices for mHealth is essential nowadays and approaches of mHealth vary from sending text messaging for medical appointment reminders to monitoring and assessing symptoms in real-time, virtually at any location via wireless networks. Interventions using mHealth have also eased medical coaching for caregivers as care partners with healthcare professionals for effective chronic disease management. Piette et al. [31] studied the comparative effectiveness of mHealth interventions supporting HF patients and their family caregivers and showed improvement for medication adherence and caregiver communication. The impact of including caregivers as a part of mHealth users—one of the care supporting groups—on actual patients’ health outcomes should continue to be studied in the context of an increasing aging society.

Moreover, long-term follow-ups of responses from patients and caregivers who have used mHealth need to be evaluated. Areas that need to be studied include the optimum length of time and frequency of the mHealth delivery system as well as type of technology and training. For example, effective frequencies of automated reminders or coaching messages, when additional reminders should be sent, and when people become tired or irritated by automated messages need to be studied. Users of mHealth might experience fatigue from automated reminders and eventually mHealth interventions could become ineffective. Other systematic reviews on chronic disease management showed that the frequency of input into mHealth systems was a burden on participants and affected the attrition rate [17]. In this review, the length of the intervention in the 12 studies varied from 2 weeks to 12 months; 5 of 12 studies (about 42%) had less than 2-month interventions while 4 studies (about 33%) had 1 year of intervention. The positive health outcomes of the various studies were not directly related to the length of the intervention or training methods of mHealth in the reviewed studies.

One aspect of mHealth approaches that also needs to be considered is effective clinical communication between patients and healthcare professionals who need to respond to patients’ questions via mHealth systems. Cingi et al. [28] reported healthcare providers (i.e., residents) expressed an improvement in communication with patients via mHealth; however, they found that using mHealth tools as the primary method of communication was strongly opposed by the healthcare providers. Vuorinen et al. [33] also reported a significant increase in the communication (i.e., telephone contacts) between nurses and patients which in turn increased the nurses’ workload during the trial. One recommendation for reducing health care providers’ workload in mHealth interventions is using advanced technology to respond to patients’ questions regarding symptoms assessed and reported via the mHealth systems.

While considering positive health outcomes (e.g., reduction of hospital readmission for HF-related conditions or medication adherence) of patients with chronic diseases, burden of healthcare professionals should be measured as an outcome of mHealth interventions. An adequate triage system can decrease healthcare professionals’ response time for emergency needs reported by mHealth users [28].

Common recommendations discussed in the studies of this systematic review to improve mHealth interventions include a simple and user-friendly-designed mHealth system, data confidentiality, lay language use for structured and automated feedback or advice, positive motivation and improving engagement [28], and inclusion of patient’s social supporters, such as family members, friends, and/or peers [31].

There are several limitations in this systematic review. First, we only selected randomized controlled trials for this review and most of them were funded studies. Therefore, the mHealth systems or smartphone apps used in the studies were validated and relatively reliable compared to health apps commercially available on the market. Literature from studies on smartphone-based interventions for chronic disease management that were small scale with or without control group and/or had a short-term follow-up has shown ambivalent results. Thus, we intended to choose robust studies that used randomization, control groups, and relevant follow-ups for outcomes. We looked at the outcome changes at different follow-up points.

Second, we did not include mHealth intervention studies for diabetes management since there is ample literature on diabetes management using mobile technology approaches [14–17]. Third, we excluded studies wherein health apps were used only by health professionals such as physicians or specialized clinical nurses. We focused on patient-centered health apps as a part of mHealth interventions in the review. Lastly, most of the reviewed studies provided smartphones or tablets to participating patients and thus, the results from this review cannot yet be generalized among those who have financial concerns regarding purchasing mHealth tools. Although the availability of wireless networks is increasing, potential mHealth users such as patients with chronic diseases or their caregivers may have limited data services for their mobile devices due to financial concerns. This might be an important issue during an emergency when patients may have no access to evidence-based medical advice via mHealth devices.

## Conclusion

The findings from the majority of reviewed studies that used mHealth interventions showed some health outcome improvement in patients with chronic disease conditions. Favorable factors in mHealth approaches are automated text reminders, frequent and accurate symptom monitoring (often in real time), and improved communication between patients and healthcare providers resulting in enhanced self-management in patients with chronic conditions. Thus, the future of mHealth is presumably optimistic. The relationship between engagement of users on mHealth tools and outcome improvement should be further studied. The studies reviewed in this paper showed disease-specific mHealth interventions that might be different from commercial mobile health apps available to the public. Rigorously tested mHealth apps developed through research should be further considered to be made available to the general population.

## Additional file

Additional file 1: Title of data: Detailed search strategy per database. Detailed search strategy per database in order to find all published interventions using mobile health applications to improve chronic disease management for adults in randomized controlled trials. (DOCX 21 kb)

## Abbreviations

CDC: The US Centers for Disease Control and Prevention
EMA: Ecological momentary assessment
HF: Heart failure
mHealth: Mobile health
PD: Parkinson’s disease
RCTs: Randomized controlled trials
UPDRS: Unified Parkinson’s Disease Rating Scale
US: The United States of America
