# Supplementary material for: Effective behavioral intervention strategies using mobile health applications for chronic disease management: a systematic review
Source: BMC Med Inform Decis Mak. 2018 Feb 20;18:12. doi: 10.1186/s12911-018-0591-0 (PMC5819153; doi:10.1186/s12911-018-0591-0)
Supplement: Supplementary file 1 — Title of data: Detailed search strategy per database. Detailed search strategy per database in order to find all published interventions using mobile health applications to improve chronic disease management for adults in randomized controlled trials. (DOCX 21 kb) [file 12911_2018_591_MOESM1_ESM.docx]

**Supplementary file 1. Detailed search strategy per database**

| **PUBMED** | | |  | |
| --- | --- | --- | --- | --- |
| **Search** | **Query** | | |  |
| #1 | ("Mobile Applications"[Mesh] OR "Cell Phones"[Mesh] OR "Computers, Handheld"[Mesh] OR "mobile health" OR "m-health" OR mhealth OR "mobile-health" OR smartphone* OR “smart-phone*” OR "mobile phone*" OR “mobile-phone*” OR “cellular phone*” OR “cellular-phone*” OR “smart device*” OR “smart-device*” OR “tablet* PC*” OR “tablet-based” OR “tablet* device*”) | | |  |
| #2 | "Disease Management"[Mesh] OR "Chronic Disease/prevention and control"[Mesh] OR "Chronic Disease/therapy"[Mesh] OR "disease* manag*" OR “disease* monitor*” OR monitor* OR “health promot*” OR Promot* | | |  |
| #3 | "Randomized Controlled Trial" [Publication Type] OR "Randomized Controlled Trials as Topic"[Mesh] OR "Controlled Clinical Trial" [Publication Type] OR randomized[Title/Abstract] OR randomised[Title/Abstract] OR randomly[Title/Abstract] OR “random* assign*”[Title/Abstract] OR trial*[Title/Abstract] | | |  |
| #4 | #1 AND #2 AND #3 | | |  |
|  |  | | |  |
| **CINAHL** | | |  | |
| **Search** | **Query** | | |  |
| #1 | (MH "Mobile Applications") | | |  |
| #2 | ("Mobile Applications"[Mesh] OR "Cell Phones"[Mesh] OR "Computers, Handheld"[Mesh] OR "mobile health" OR "m-health" OR mhealth OR "mobile-health" OR smartphone* OR “smart-phone*” OR "mobile phone*" OR “mobile-phone*” OR “cellular phone*” OR “cellular-phone*” OR “smart device*” OR “smart-device*” OR “tablet* PC*” OR “tablet-based” OR “tablet* device*”) | | |  |
| #3 | #1 OR #2 | | |  |
| #4 | (MM "Disease Management") | | |  |
| #5 | "Disease Management"[Mesh] OR "Chronic Disease/prevention and control"[Mesh] OR "Chronic Disease/therapy"[Mesh] OR "disease* manag*" OR “disease* monitor*” OR monitor* OR “health promot*” OR Promot* | | |  |
| #6 | #4 OR #5 | | |  |
| #7 | (MM "Randomized Controlled Trials") OR (MM "Random Sample+") OR (MM "Random Assignment") OR (MM "Stratified Random Sample") OR (MM "Systematic Random Sample") OR (MM "Simple Random Sample") | | |  |
| #8 | TI random* OR AB random* | | |  |
| #9 | #7 OR #8 | | |  |
| #10 | #3 AND #6 AND #9 | | |  |
|  |  | | |  |
| **Cochrane Library** | | | | |
| **Search** | **Query** | | |  |
| #1 | MeSH descriptor: [Mobile Applications] explode all trees OR MeSH descriptor: [Cell Phones] explode all trees OR MeSH descriptor: [Computers, Handheld] explode all trees OR "mobile health" or "m-health" or mhealth or "mobile-health" or smartphone* or "smart-phone*" or "mobile phone*" or "mobile-phone*" or "cellular phone*" or "cellular-phone*" or "smart device*" or "smart-device*" or "tablet* PC*" or "tablet-based" or "tablet* device*" | | |  |
| #2 | MeSH descriptor: [Disease Management] explode all trees OR MeSH descriptor: [Chronic Disease] explode all trees OR "disease* manag*" OR "disease* monitor*" OR monitor* OR "health promot*" OR Promot* | | |  |
| #3 | #1 AND #2 | | |  |
|  |  | | |  |
| **EMBASE** | | |  | |
| **Search** | **Query** | | |  |
| #1 | mobile phone'/exp/mj OR 'mobile application'/exp/mj OR 'mobile application' OR 'mobile' | | |  |
| #2 | 'disease management'/exp/mj OR 'disease management' | | |  |
| #3 | randomization'/exp/mj OR 'randomization' OR 'random sample'/exp/mj OR 'random' | | |  |
| #4 | #1 AND #2 AND #3 | | |  |
|  |  | | |  |
| **PsycINFO** | | |  | |
| **Search** | **Query** | | |  |
| #1 | SU.EXACT.EXPLODE("Mobile Devices") OR SU.EXACT.EXPLODE("Cellular Phones") OR "Mobile Application*" OR "mobile health" OR "m-health" OR "mhealth" OR "mobile-health" OR smartphone* OR "smart-phone*" OR "mobile phone*" OR "mobile-phone*" OR "cellular phone*" OR "cellular-phone*" OR "smart device*" OR "smart-device*" OR "tablet* PC*" OR "tablet-based" OR "tablet* device*" | | |  |
| #2 | "Disease Management" [Mesh] OR "Chronic Disease/prevention and control" [Mesh] OR "Chronic Disease/therapy" [Mesh] OR "disease* manag*" OR "disease* monitor*" OR monitor* OR "health promot*" OR Promot* | | |  |
| #3 | random* | | |  |
| #4 | (SU.EXACT.EXPLODE("Mobile Devices") OR SU.EXACT.EXPLODE("Cellular Phones") OR "Mobile Application*" OR "mobile health" OR "m-health" OR mhealth OR "mobile-health" OR smartphone* OR "smart-phone*" OR "mobile phone*" OR "mobile-phone*" OR "cellular phone*" OR "cellular-phone*" OR "smart device*" OR "smart-device*" OR "tablet* PC*" OR "tablet-based" OR "tablet* device*") AND ("Disease Management" [Mesh] OR "Chronic Disease/prevention and control" [Mesh] OR "Chronic Disease/therapy" [Mesh] OR "disease* manag*" OR "disease* monitor*" OR monitor* OR "health promot*" OR Promot*) AND random* | | |  |
|  | |  |  | |
| **Web of Science** | | | | |
| **Search** | **Query** | | |  |
| #1 | TS= ("Mobile Application*") OR TS= ("Cell phone*") OR TS= ("Handheld Computer*") OR TS= ("mobile health") OR TS= ("m-health") OR TS= (mhealth) OR TS= ("mobile-health") OR TS= (smartphone*) OR TS= (smart-phone*) OR TS= ("mobile phone*") OR TS= ("mobile-phone*") OR TS= ("cellular phone*") OR TS= ("smart device*") OR TS= ("tablet* PC*") OR TS= ("tablet-based") OR TS= ("tablet* device*") | | |  |
| #2 | TS= ("Disease Management" [Mesh] OR "Chronic Disease/prevention and control" [Mesh] OR "Chronic Disease/therapy" [Mesh] OR "disease* manag*" OR "disease* monitor*" OR monitor* OR "health promot*" OR Promot*) | | |  |
| #3 | TI= (randomised) OR TI= (randomized) OR TI= (randomly) OR TI= ("random* assign*") OR TI= (trial*) OR TS= (random*) | | |  |
| #4 | #1 AND #2 AND #3 | | |  |
